# New endoscopic procedure for bladder wall closure: results from the porcine model

**DOI:** 10.1038/s41598-019-54304-w

**Published:** 2019-12-10

**Authors:** Carlos Oliveira, Alexandre A. Barros, Rui L. Reis, Jorge Correia-Pinto, Estêvão Lima

**Affiliations:** 1 Life and Health Sciences Research Institute, School of Medicine, University of Minho, Braga, Portugal; 2 ICVS/3B’s, PT Government Associate Laboratory, Braga, Guimarães Portugal; 3 Department of Urology, Hospital de Braga, Braga, Portugal; 4 3B´s Research Group – Biomaterials, Biodegradables and Biomimetics, University of Minho, Headquarters of the European Institute of Excellence on Tissue Engineering and Regenerative Medicine, Avepark – Parque de Ciência e Tecnologia, 4805-017 Barco, GMR Portugal

**Keywords:** Bladder, Ureter

## Abstract

Upper urinary tract urothelial carcinomas are usually managed by radical nephroureterectomy (RNU), often followed by intravesical chemotherapy to minimize recurrence. Open surgery is the gold standard procedure for RNU, but it associates with high morbidity, and it has been increasingly replaced by minimally invasive strategies, such as laparoscopy and endoscopy. Although effective, endoscopic ureteral excision leaves the bladder unsutured, increasing the risk of tumor spillage, and precluding the immediate administration of intravesical chemotherapy. Here we describe a new method to close the bladder wall after ureteral excision, using barbed sutures via the endoscopic access. Our results in 8 female pigs demonstrate that this method is effective to close the bladder wall. The procedure was completed in a median time of 24 min, and no adverse events were registered in the follow-up or at the three-week necropsy. This technique improves a previous approach described by our group because the device is more flexible and allows to tie the knots inside the bladder. Barbed sutures have been used in the clinical practice for other types of surgeries, and therefore this method can further be adapted to human patients with no safety concerns. Its use may allow to administer intravesical chemotherapy, which reduces tumor recurrence and improves patient outcomes.

## Introduction

Urothelial carcinomas are frequent tumors located in the urinary tract^1^. When located in the upper urinary tract (5–7% of cases^2^) they are frequently aggressive, and have high post-treatment recurrence rates (22–47%)^1^.

Treatment for upper urinary tract urothelial carcinomas is radical nephroureterectomy (RNU). In this procedure, the affected kidney, ureter, and a bladder cuff around the ureter are removed. The standard method for RNU in high-risk upper urinary tract carcinomas, is open surgery^1,3,4^ with two incisions: one in the flank to access the kidney and other in the lower abdomen to access the distal ureter and its orifice, and suture the bladder wall. Given the high morbidity of this procedure, laparoscopic and robotic RNU are increasingly replacing open surgery without compromising oncologic outcomes and survival rates^5–7^.

Independently of the technique used, a critical step of the surgery is the management of the distal ureter and the bladder cuff. Endoscopy may be performed to excise the bladder cuff. In this technique, an endoscopic-guided hook electrode incises a circumference of bladder mucosa around the ureteral orifice, deep to the perivesical fat level. After detaching the ureter, kidney and ureter are excised through the laparoscopic access and the bladder is often catheterized without closing the defect^3,6,8^.

To prevent tumor spillage, the bladder wall should be sutured as early as possible after ureteral excision. Evidence shows that post-operative intravesical chemotherapy reduces the risk of bladder tumor recurrence. A single dose of intravesical mitomycin C or pirarubicin reduces bladder tumor recurrence in the first year post-RNU^9–11^, more effectively when administered within the first 6 to 72 h after RNU^12^. However, chemotherapy administration also requires an adequately closed bladder wall, since chemotherapy leakage might cause potentially lethal effects.

Our group previously reported an endoscopic technique for bladder closure, using an adapted laparoscopic suturing machine^13,14^. In this technique, the large diameter and rigid structure of the device forced to enlarge the urethral access and pull out the strings to tie the knots. Therefore, although effective in the pig model, this technique had limited human applications.

In this study, we aim to develop an endoscopic method to close the bladder wall that can be applied to human patients submitted to open or laparoscopic RNU. We present a new technique using barbed sutures, developed in the pig model, and discuss its advantages for human application.

## Materials and Methods

### Ethical statement

This study describes feasibility of an endoscopic procedure in an animal model. The study followed the internal protocol for animal experiments, and was approved by the ethics subcommittee for Life and Health Sciences of University of Minho (017/2019).

### Study design and experimental animals

Proof-of concept experiment and training for the procedure was first done in a female pig which had been used for previous studies and was about to be sacrificed. The endoscopic procedure was controlled by open surgery to verify bladder closure. To test for leaks, the bladder was filled with a saline solution of 3% methylene blue until fully distended.

Laparoscopic RNU with the new technique for endoscopic bladder closure was afterwards tested in eight female pigs (*Sus scrofus domesticus*), with 25 to 30 kg. Animals were kept in single cages before and after intervention. The did not have any food or water in the previous 12 hours. Prophylactic antibiotic cephalosporin was administered to each animal one hour before, and 24 hours after the procedure. During the procedure, they were under general anesthesia and mechanical ventilation, as described elsewhere^14^.

The same surgeon with experience in endoscopic surgery completed the procedure in all animals.

### Endoscopic bladder closure

#### Access

A 0.035-inch flexible tip guidewire was inserted through the urethra with the help of a rigid ureteroscope (Karl Storz,Tuttlingen, Germany), which was later removed. An urethral dilator was inserted to allow introducing a 21-F rigid cystoscope.

#### Incision

**Figure 1:**
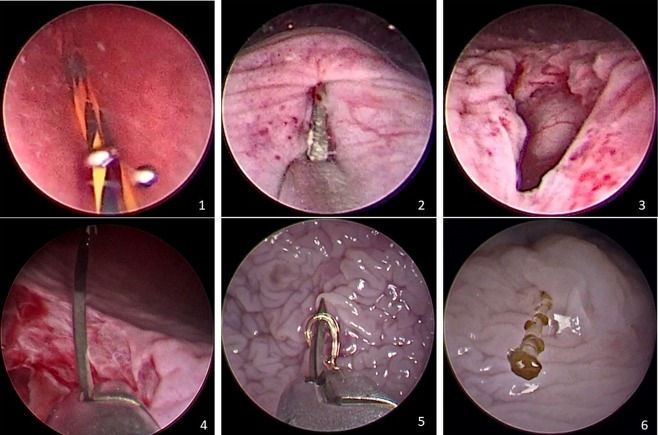
Surgical steps: 1. Access with flexible guidewire; 2. Scissor Incision; 3. View of the bladder defect; 4. Suture with needle holder; 5. Passing the tip of the needle through the end loop; 6. Final result.

**Figure 2:**
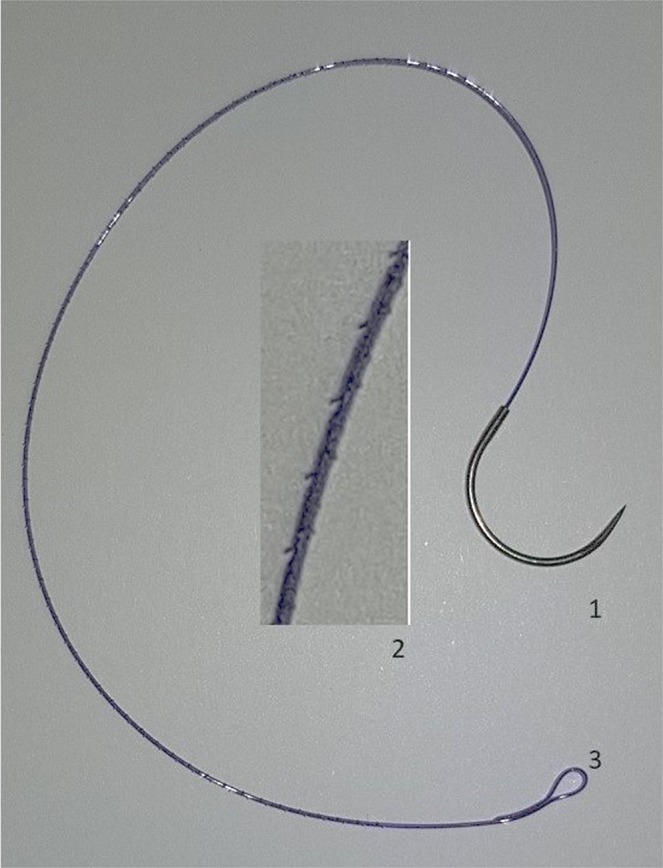
V-locTM90 suture: 1- needle, 2- Ampliation to show the anchoring projections on the surface of the suture, 3- blind loop at the distal part of the suture.

**Figure 3:**
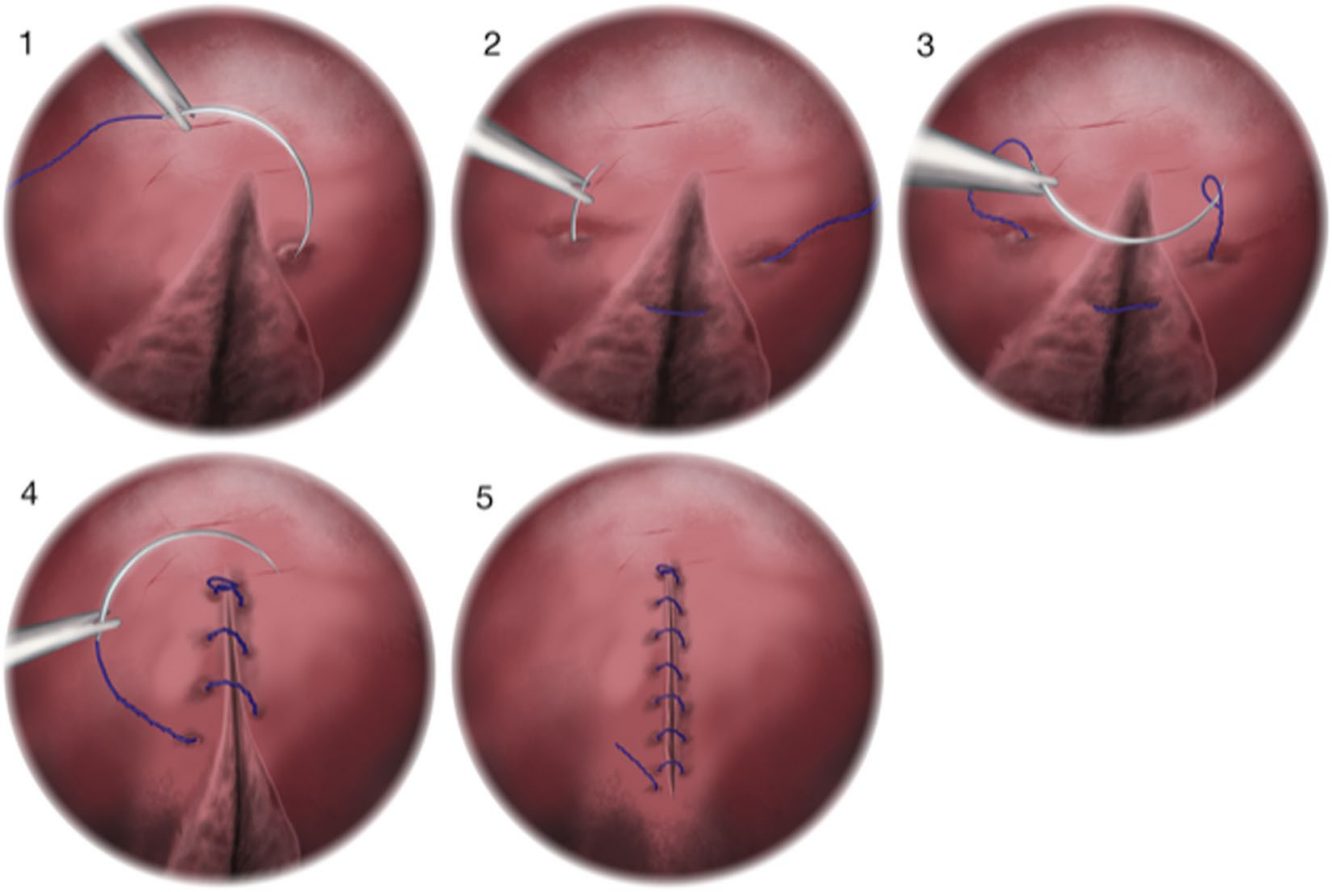
Illustration of the steps (1–5) to close bladder incision using barbed sutures Illustration kindly provided by Ana Goios.

A full-thickness circular incision was done around the ureteral bladder orifice, with endoscopic scissors (26168A; Karl Storz, Tuttlingen, Germany) introduced through the working channel of the cystoscope (Figs. 1, 1–3).

#### Suture

The bladder incision was closed using the V-Loc^TM^90 Absorbable Suture, currently used in the clinical practice to close other types of wounds. This barbed suture device has a semi-circumferential needle, small projections along the wire that help anchoring the knots, and a blind loop in the distal part (Fig. 2).

The 21-F cystoscope was replaced by a 26-F nephroscope. Using a 3 mm laparoscopic needle holder, the V-Loc suture was inserted through the working channel of the nephroscope. The needle was passed through the edges on each side of the incision, and through the loop at the end of the barbed suture. The thread was pulled progressively, making additional stitches to join the incision edges. With the barbed wire holding the suture, excess thread was cut and removed, with the needle, through the working channel of the nephroscope (Fig. 1, 4–6; Fig. 3, 1–5). The kidney and detached ureter were excised by standard laparoscopic RNU.

#### Post-operative procedures and follow-up

Animals were left uncatheterized, assessed daily for three weeks, and controlled by cystoscopy one week after the procedure. At the end of follow-up, they were sacrificed and necropsied to assess healing quality and inspect for bowel adherences, peritonitis or wound dehiscence.

### Outcomes

For each animal, we reported: (a) time to perform the procedure, from end of bladder incision to end of bladder closure; (b) postoperative events; (c) 1-week cystoscopy findings; (d) necropsy findings.

Time summary statistics is presented by median and range.

## Results

The first proof-of-concept experiment was completed successfully. The bladder was adequately closed and no leakage was detected in the methylene blue test. Kidney and distal ureter with bladder cuff were removed en bloc (Supplementary Fig. 1).

We completed the procedure in all 8 animals, with precisely placed sutures and no complications, in a median time of 24 min (range: 17 min 45 sec – 30 min and 10 sec). A surgeon with experience in endoscopic surgery completed the procedure easily, not requiring a steep learning curve.

Pigs urinated normally and tolerated a regular diet from the morning after surgery. No complications or post-operative events were registered. During follow-up, the animals ambulated freely, with normal behavior and no adverse events. Control cystoscopy after one week showed a closed bladder wall and no dehiscence in all animals. After 3 weeks, necropsy confirmed healed bladder wall incisions with suture remnants but no evidence of transmural dehiscence or calcification. The peritoneal cavities of the animals were not infected or adhered (Table 1).

**Table 1:** Time and outcomes of the procedure in the 8 female pigs tested.

Specimen	Procedure time (min:sec)	1-week cystoscopy findings	Necropsy findings
1	23:30	Closed bladder. No signs of dehiscence.	Normal closure. Minor inflamation
2	27:00	Closed bladder. No signs of dehiscence.	Normal closure
3	20:12	Closed bladder. No signs of dehiscence.	Normal closure
4	17:45	Closed bladder. No signs of dehiscence.	Normal closure
5	22:27	Closed bladder. No signs of dehiscence.	Normal closure
6	30:10	Closed bladder. No signs of dehiscence.	Normal closure
7	24:55	Closed bladder. No signs of dehiscence.	Normal closure
8	26:05	Closed bladder. No signs of dehiscence.	Normal closure

## Discussion

In the era of minimally invasive surgery, laparoscopic RNU has been increasingly replacing the gold standard open surgery^5–7^. However, laparoscopic access to the kidney difficults managing the ureteral bladder cuff. Several methods have been used to detach the distal ureter while avoiding additional incisions. Endoscopy is the least invasive of these methods, but present techniques leave the bladder incision unsutured and require catheterization^3,6,8^. This precludes an immediate administration of intravesical chemotherapy, which has been recommended for maximal efficacy in reducing tumor recurrence^12^. Here we describe a new endoscopic technique to close the bladder wall after detaching the ureter. We demonstrate that, in the pig model, this technique is easy, reproducible, and safe.

The technique here described uses barbed sutures to close the bladder wall through the endoscopic access, and thus avoids a second incision. The sutures were effective in holding the suture, as verified through follow-up cystoscopy and necropsy. Since the bladder was adequately closed in all animals, with no adverse events or dehiscence, it is safe to conclude that the risk of tumor spillage into the urinary tract is reduced, and that chemotherapy can be administered in the immediate post-operative period with no risk of leakage to the peritoneal cavity.

Our group had previously reported an approach to this procedure, using a rigid laparoscopic suturing machine^13,14^. The present methodology improves the previous approach^13,14^, because it uses a more flexible and smaller diameter device, and can thus be applied it to female or male humans patients. Moreover, the present strategy allows tying the knots inside the bladder, which simplifies the procedure and reduces infection risks. Surgeons experienced in endoscopy can easily learn this technique, which only requires specific training in tying the knots internally. The new procedure is derived from devices already in use in the clinical practice, therefore it raises no ethical concerns or specific clinical trials for validation. This technique may be further explored for other applications, such as for trans-cytoscopic excision of bladder tumors followed by primary repair.

### Limitations

Despite the advantages of the pig as an animal model for surgical procedures, its urinary system differs from the human in several aspects and particularly in size, limiting endoscopic manipulations. Moreover, the pig bladder is more mobile and difficult to pierce. Thus, we anticipate that the technique will be easier in human patients than in pigs.

The procedure also presented some limitations, specifically a difficult visualization. Since the needle holder was manipulated through the working channel of the nephroscope, the range of movements of the needle holder was limited to the width of the working channel, and the camera and needle holder had to be manipulated in block.

We also recognize some limitations on this study. The procedure was tested in a convenience sample of 8 animals, which may be considered a small sample. However, the study did not require statistical inferences, and this number resulted from a compromise with the ethical concerns of animal testing. We trust that this should not preclude from applying the technique to human patients because: (a) this can be considered an adaptation of previously existing methodologies and (b) in all animals the bladder was adequately closed with no adverse events.

Finally, only one surgeon did the experiment in all animals, and therefore we cannot infer how other professionals will adapt to the technique. However, the fact that, since the first experiment, all instances had very positive results, gives confidence that other surgeons with experience in endoscopy will easily learn it.

## Conclusions

We present a technique to close the bladder wall in laparoscopic RNU, using barbed sutures, via the endoscopic access. It improves previous methodologies, since it maintains a minimally invasive procedure but allows to administer chemotherapy in the hours following the surgery, with no risk of peritoneal leakage. This technique was fast, reproducible and safe in the pig model. It can be applied to human patients because it is based on devices already in use in the clinical practice. If human experiments confirm its feasibility this could contribute to improve the outcomes of oncologic patients subject to RNU.

## Supplementary information

Suplementary Images

Endoscopic Bladder wall suture
