# Supplementary material for: New endoscopic procedure for bladder wall closure: results from the porcine model
Source: Sci Rep. 2019 Dec 10;9:18747. doi: 10.1038/s41598-019-54304-w (PMC6904675; doi:10.1038/s41598-019-54304-w)

New endoscopic procedure for bladder wall closure: results from the porcine model.

Carlos Oliveira, Alexandre A. Barros, Rui L. Reis, Jorge Correia-Pinto,
Estêvão Lima

Supplementary Images

Supplementary figure 1: Surgical specimen removed in the procedure.

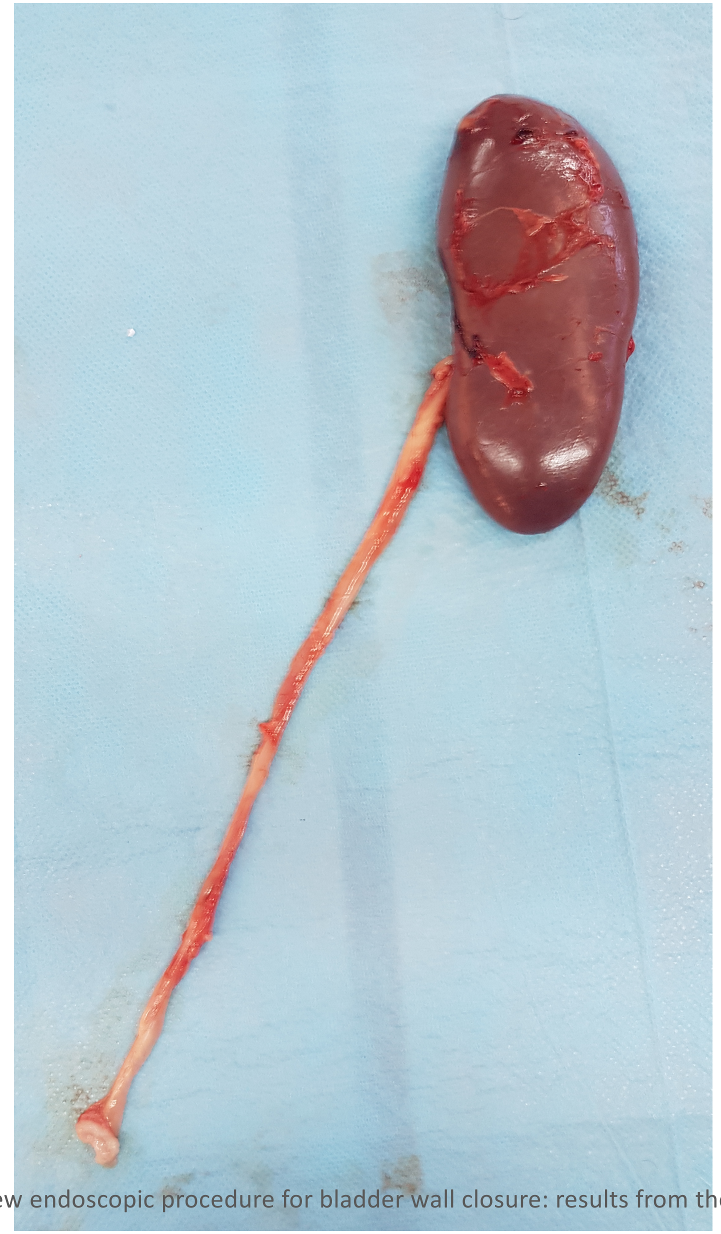

Oliveira et al. New endoscopic procedure for bladder wall closure: results from the porcine model.

Supplementary figure 2:
Ampliation of the distal ureter with bladder cuff

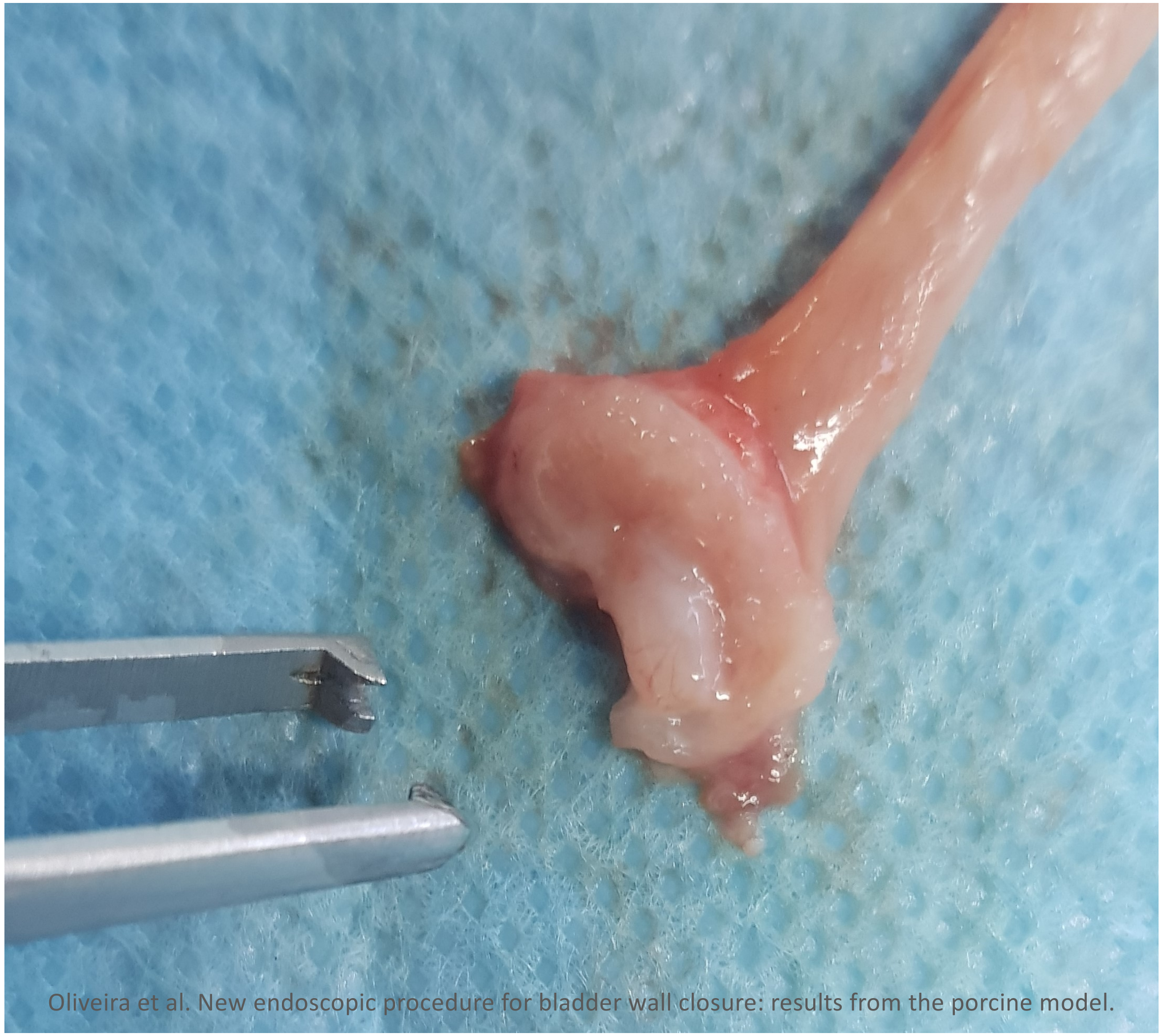

Supplement: Supplementary file 1 — Suplementary Images [file 41598_2019_54304_MOESM1_ESM.pdf]
